# Rumen microbiome-driven insight into bile acid metabolism and host metabolic regulation

**DOI:** 10.1093/ismejo/wrae098

**Published:** 2024-06-05

**Authors:** Boyan Zhang, Xianzhe Jiang, Yue Yu, Yimeng Cui, Wei Wang, Hailing Luo, Sokratis Stergiadis, Bing Wang

**Affiliations:** State Key Laboratory of Animal Nutrition and Feeding, College of Animal Science and Technology, China Agricultural University, Beijing 100193, P. R. China; State Key Laboratory of Animal Nutrition and Feeding, College of Animal Science and Technology, China Agricultural University, Beijing 100193, P. R. China; State Key Laboratory of Animal Nutrition and Feeding, College of Animal Science and Technology, China Agricultural University, Beijing 100193, P. R. China; State Key Laboratory of Animal Nutrition and Feeding, College of Animal Science and Technology, China Agricultural University, Beijing 100193, P. R. China; State Key Laboratory of Animal Nutrition and Feeding, College of Animal Science and Technology, China Agricultural University, Beijing 100193, P. R. China; State Key Laboratory of Animal Nutrition and Feeding, College of Animal Science and Technology, China Agricultural University, Beijing 100193, P. R. China; Department of Animal Sciences, School of Agriculture Policy and Development, University of Reading, Reading RG6 6EU, United Kingdom; State Key Laboratory of Animal Nutrition and Feeding, College of Animal Science and Technology, China Agricultural University, Beijing 100193, P. R. China

**Keywords:** bile acids, microbial biotransformation, rumen microbiome, metagenome-assembled genomes, metabolic regulation

## Abstract

Gut microbes play a crucial role in transforming primary bile acids (BAs) into secondary forms, which influence systemic metabolic processes. The rumen, a distinctive and critical microbial habitat in ruminants, boasts a diverse array of microbial species with multifaceted metabolic capabilities. There remains a gap in our understanding of BA metabolism within this ecosystem. Herein, through the analysis of 9371 metagenome-assembled genomes and 329 cultured organisms from the rumen, we identified two enzymes integral to BA metabolism: 3-dehydro-bile acid delta4,6-reductase (*baiN*) and the bile acid:Na + symporter family (*BASS*). Both *in vitro* and *in vivo* experiments were employed by introducing exogenous BAs. We revealed a transformation of BAs in rumen and found an enzyme cluster, including L-ribulose-5-phosphate 3-epimerase and dihydroorotate dehydrogenase. This cluster, distinct from the previously known BA-inducible operon responsible for 7α-dehydroxylation, suggests a previously unrecognized pathway potentially converting primary BAs into secondary BAs. Moreover, our *in vivo* experiments indicated that microbial BA administration in the rumen can modulate amino acid and lipid metabolism, with systemic impacts underscored by core secondary BAs and their metabolites. Our study provides insights into the rumen microbiome’s role in BA metabolism, revealing a complex microbial pathway for BA biotransformation and its subsequent effect on host metabolic pathways, including those for glucose, amino acids, and lipids. This research not only advances our understanding of microbial BA metabolism but also underscores its wider implications for metabolic regulation, offering opportunities for improving animal and potentially human health.

## Graphical Abstract

**Graphical Abstract:**
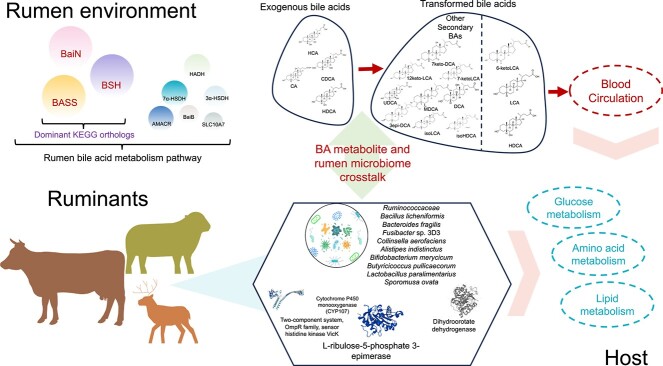

## Introduction

Bile acids (BAs), originating both from host synthesis and microbial metabolism, are vital metabolites that contribute to gut health and stability by regulating microbe-host interactions within the intestinal ecosystem [1, 2]. The influence of BA-mediated gut microbiota extends to shaping host physiology, encompassing the regulation of metabolic evolution, immune responses, and the coordination of intricate host activities [3]. The essential role of BAs in the health and nutrition of humans and animals has received increasing attention, particularly in light of their pivotal microbial deconjugation and biotransformation from primary BAs to secondary BAs by gut microbes [4, 5]. Microbial transformations in the lower gut are critical in modifying BA metabolism and shaping gut microbial community structure and function [6]. The enzymatic conversion of primary BAs to secondary BAs alters their structure and receptor binding affinity within intestinal, hepatic, and systemic tissues, consequently impacting tissue homeostasis [6]. The bacterial BA-inducible (bai) operon plays a crucial role in the dehydroxylation and epimerization processes. This operon includes C7 hydroxyl dehydroxylation of cholic acid (CA) and chenodeoxycholic acid (CDCA), resulting in the production of deoxycholic acid (DCA) and lithocholic acid (LCA), respectively [2]. In particular, both primary and secondary BAs collectively serve as a habitat filter, augmenting colonization resistance [7].

The rumen, a distinctive organ in ruminants often conceptualized as a biological “black box,” exerts a more substantial influence on host physiology than the small intestine and contains an exceptionally diverse microbial community that endows its host with specialized metabolic capabilities [8], such as specialized chemical synthesis, detoxification of plant-derived toxins, and modulation of host immune homeostasis [9]. The rumen’s intricate structure, coupled with its anaerobic conditions and continuous exposure to a wide range of dietary substrates, suggests it may harbor yet-undiscovered bacteria and functional genes involved in BA metabolism, although BAs are not normally found in the rumen under physiological conditions. BAs and their derivatives enhance nutrient absorption, regulate lipid and energy metabolism, and serve as potential treatments for inflammatory metabolic diseases [10]. Additionally, exogenous BAs have been widely utilized as feed additives across various species, including pigs, chickens, and fish, due to their effects on nutrient absorption and metabolism [11–14]. Hyodeoxycholic acid (HDCA) has shown promising results in modulating the gut-liver axis, thereby alleviating non-alcoholic fatty liver disease, and this specific BA has demonstrated therapeutic potential in multiple mouse models [15]. Our recent findings indicate that supplementing exogenous BAs in the rumen significantly affects lipid regulation in lambs, reducing lipid deposition in the backfat and tail [16]. Consequently, given the bioactive properties of BAs, we hypothesize that they might profoundly influence the rumen microbiome and, subsequently, overall host metabolism.

Unraveling the BA-microbiome metabolic relationship can identify pathways associated with functional newly microbial BA production [4, 5] and metabolic health in humans and animals [4, 5]. It is possible to investigate the relationship between the rumen microbiome and BAs and to explore the BA-associated rumen microbiome dynamics. We started by analyzing rumen metagenome-assembled genomes (MAGs), and we then performed *in vitro* and *in vivo* anaerobic fermentation to identify the impact of introducing exogenous BAs on the rumen microbiome. Then, by employing integrative targeted serum metabolomics, we explained the contribution of rumen microbial-derived BAs to systemic BA circulation and their role in enhancing host metabolism. Collectively, our study provides a possible mechanistic explanation for the BA-associated rumen microbiome as a driver of BA metabolism.

## Materials and methods

The workflow overview of the methods employed in this study is presented in Supplementary Fig. S1. All animal procedures in the present study were approved by the Animal Care Committee of China Agricultural University (Beijing, China; approval no. AW30901202-1–1).

### High-quality ruminal MAGs re-analysis

We retrieved raw data from 9371 rumen MAGs across six studies [17–22]. Integration of the obtained MAGs was achieved using the DAS tool (v.1.1.1) [23]. The completeness and contamination levels of prokaryotic MAGs were assessed using CheckM (v.1.0.7) [24], with quality scores defined as −5 × contamination [25]. Use MAGs with >80% integrity and <10% contamination for downstream analysis. High-quality MAGs are dereplicated using the dRep software [26]. After filtering, rumen MAGs underwent annotation using GTDB-Tk [27] (v.0.1.6) based on the Genome Taxonomy Database. We used KofamScan (v.1.1.0) to give *K* numbers to the MAGs’ protein sequences by comparing them with KOfam, a specialized database based on KOs. We considered the KO assignments with scores above the default threshold and *E* values within the required range for KOs as the most reliable. We then connected these results to KEGG pathways and EC numbers for better interpretation. Initially, 82 KOs were identified as BA metabolism-associated KEGG orthologs (BAKOs), and from these, 15 common KOs related to BA metabolism were selected based on previous research [4, 5]. For the phylogenetic analysis, we used PhyloPhlAn (v.1.0) to build a maximum-likelihood phylogenomic tree and tvBOT for visualization [28]. Following the same process, we annotated high-quality ruminal MAGs from rumen sample metagenomes collected in this study.

### IMG/G rumen cultured organisms and functional gene annotation

To annotate BA metabolism genes in isolated and cultured rumen microorganisms, we used the “Genomes by Ecosystem” module search under the “Genome Browser” function of the Integrated Microbial Genomes (IMG) system to search for keywords in the “Specific Ecosystem” category microorganisms associated with “rumen” [29]. Select all microorganisms under the “Isolates” category in the search results and download all microbial annotation data, including microbial integrity, sequencing results, KO annotations, and other information. Search and filter 82 BAKOs for each downloaded microorganism information to find microorganisms containing 82 BAKOs.

### *In vitro and in vivo* rumen fermentation

Rumen fluid was collected from six Hu sheep, which had fistulas and were ~6 months old. Prior to the morning feeding, mixed rumen contents were collected and filtered through four layers of cheesecloth to get the filtered rumen fluid into a graduated cylinder. Throughout the entire process, CO_2_ was continuously injected. The buffer, with a pH of 6.87, was prepared following the previous method [30], and CO_2_ was continuously injected into the buffer for ~30 min before inoculation. Cysteine hydrochloride was incorporated into the buffer as a chemical reducing agent. For each incubation glass bottle (with a capacity of 120 ml), 0.5 g of substrates, 25 ml of filtered rumen fluid, and 50 ml of pre-heated buffer solution were added. The rumen fluid was divided into 28 bottles, categorized into four groups: (i) control group (no treatment, C); (ii) 8 mg group (8 mg BAs added, CA); (iii) 16 mg group (16 mg BAs added, CB); and (iv) 32 mg group (32 mg BAs added, CC). The exogenous supplemental BAs were derived from swine [three primary BAs: 80.7% hyocholic acid (HCA), 12.7% CDCA, and 1.2% CA; one secondary BA: 4.4% HDCA; two conjugated BAs: 0.6% taurochenodeoxycholic acid and 0.4% taurocholic acid]. To establish an anaerobic condition, all bottles were purged with N_2_, sealed rapidly with butyl rubber stoppers and Hungate’s screw caps, and then immediately connected to the AGRS-III equipment using medical transfusion tubes. The *in vitro* experiments, conducted three times over a period of 2 weeks, demonstrated consistent gas production trends across trials, prompting the selection of samples from the third trial for subsequent analysis.

Twelve Tan-lambs (*Ovis aries*), ~6 months, with an average bodyweight of 25 kg, were selected. The exogenous BAs utilized were consistent with those employed in the *in vitro* study. The lambs were randomly assigned to two dietary treatment groups: a control group (C-vivo) and a group receiving a diet supplemented with 0.04% exogenous mixed BAs (on a dry matter basis, designated as BA-vivo). There were six animals in each group. The animal variables resulting from BA feeding have been previously reported in our published research [16]. The specific ingredients and nutritional composition of the diet are provided to the animals in both *in vitro* and *in vivo* studies (Supplementary Table S1a and b).

### Sampling scheme and rumen fermentation characteristics analysis

Cumulative gas production was continuously monitored in real-time using the automated trace gas recording system (AGRS-III, Beijing, China), and the pH was measured using a German Testo 205 pH meter at the end of each fermentation. Subsequently, 2 ml of filtered culture fluid was sampled into DNase-free polypropylene tubes and stored at −80°C for subsequent analysis. For the *in vivo* study, blood was collected from the jugular vein of the animal, 6 hr after the morning feeding and centrifuged for 10 min at 3000 × *g* to separate serum. Rumen fluid was obtained from the ventral part of the rumen after slaughtering, prior to morning feeding, by straining the ruminal content through four layers of cheesecloth. All collected samples were then stored in liquid nitrogen for subsequent analysis. The pH of the rumen fluid was promptly measured using a German Testo 205 pH meter, calibrated before use with automatic temperature compensation. The volatile fatty acid (VFA) concentrations were determined using gas chromatography (Trace 1300; Thermo Fisher Scientific Co., Ltd, Shanghai, China). Ammonia-N levels were determined using previously published methods [31].

### Rumen quantitative bile acid metabolomics analysis

Rumen fluid and serum BAs were quantified using the previous procedure [32]. UHPLC-QE Orbitrap/MS (Vanquish, Thermo Fisher Scientific), equipped with a Waters ACQUITY UPLC BEH C18 column (150^*^2.1 mm, 1.7 μm, Waters), was used. An Orbitrap Exploris 120 mass spectrometer (Thermo Fisher Scientific) was used for assay development. The details of the UHPLC-QE Orbitrap/MS separation and analysis conditions can be found in the previous study [32]. The sample concentration is determined by multiplying the calculated concentration by the dilution factor. The concentration of the target metabolite, denoted as *C_M_* (metabolite concentration, nmol/l), in the sample is calculated as the product of the final measured concentration (C*_F_*) of the sample and the final volume (diluted volume) *V_F_* (μl) of the sample, divided by the sample volume *V_S_* (μl)


$$ {c}_M\left[\mathrm{nmol}\cdot{l}^{-1}\right]=\frac{c_F\left[\mathrm{nmol}\cdot{l}^{-1}\right]\cdot{V}_F\left[\mu l\right]}{V_s\left[\mu l\right]}. $$

In accordance with the definition of primary and secondary BA, considering whether they undergo transformation and modification by microorganisms and referencing a prior study [5], the following BAs are classified: primary BAs: CDCA, 3β-CA, ω-muricholic acid (ω-MCA), α-MCA, β-MCA, HCA, CA, CDCA-3-sulfate, and GCA-3-sulfate; secondary BAs: isoLCA, LCA, 7-ketoLCA, 12-ketoLCA, murideoxycholic acid (MDCA), isoUDCA, isoHDCA, ursodeoxycholic acid (UDCA), HDCA, 3-epideoxycholic acid (3-epiDCA), DCA, 7,12-diketoLCA, 6,7-diketoLCA, 7-ketoDCA, allocholic acid (alloCA), UDCA-3-sulfate, and glycolithocholic acid-3-sulfate (GlyLCA-3-sulfate); not classified as primary or secondary BAs: dehydrolithocholic acid (DHLCA), 6-ketoLCA, dehydrocholic acid (DHCA), 12-DHCA, 3-DHCA, ursocholic acid (UCA), and GlyDHCA. These classifications are based on whether the BAs are considered primary (originating directly from the liver) or secondary (formed through microbial transformation in the intestine).

### Metagenomic sequencing and construction of non-redundant genes set

Total DNA was extracted from each ruminal content sample (~200 mg per sample) using a microbead stirrer (Biospec Products, Bartlesville, OK, USA) in accordance with a previously established method [33]. The integrity of the extracted DNA was assessed through electrophoresis on 0.8% agarose gels, and DNA quantity and quality were determined using a Nanodrop ND-1000 (Thermo Fisher Scientific, Waltman, MA, USA). Subsequently, high-quality DNA from each sample was employed to construct a metagenomic library with an insert size of 350 bp, adhering to the manufacturer’s instructions for the TruSeq DNA PCR-Free Library Preparation Kit (Illumina, San Diego, CA, USA). Subsequently, the library underwent sequencing on the NovaSeq 6000 platform (Illumina, San Diego, CA, USA).

Sequence data from both *in vitro* and *in vivo* rumen microbiomes underwent quality filtering with Fastp (v0.20.0) (https://github.com/OpenGene/fastp) to eliminate sequencing adapters. Additionally, bowtie2 (v.0.7.17) [34] was employed to remove host, food, and human sequences. Assembly of high-quality reads from each sample was carried out using MEGAHIT [35] (v.1.1.1) and QUAST [36]. MetaGeneMark [37] software (http://exon.gatech.edu/meta_gmhmmp.cgi) with default parameters was utilized to identify coding regions of the genome. Redundancy was then eliminated using MMseqs2 [38] software (https://github.com/soedinglab/mmseqs2) with a similarity threshold set at 95% and a coverage threshold set at 90%.

Taxonomic assessment of the rumen microbiota employed DIAMOND against the RefSeq non-redundant proteins (http://www.ncbi.nlm.nih.gov/RefSeq/) [39]. Taxonomic profiles included domain, phylum, genus, and species levels, with relative abundances calculated. Principal coordinates analysis (PCoA) based on Bray–Curtis dissimilarity matrices at the species level was performed. Contigs were annotated using DIAMOND against the KEGG database (http://www.genome.jp/kegg/) with an *E* value of 1e-5. The CAZy annotation was conducted using USEARCH (http://www.drive5.com/usearch/). The annotation of microbial cytochrome P450 monooxygenase was conducted based on the Cytochrome P450 Engineering Database version 6.0 (https://cyped.biocatnet.de/) [40]. The metagenomic sequencing data obtained in this study were further used for MAGs research, and the remaining high-quality contigs were binned into MAGs using three different approaches with default parameters: MaxBin [41] (v.2.2.4), MetaBAT2 [42] (v.2.11.1), and CONCOCT [43] (v.0.4.0). The MAG assembly results obtained from different metagenomes were integrated using the DAS tool [23] (v.1.1.1). The remaining analysis steps are the same as above.

### Quantitative serum metabolomics analysis

We conducted 600 Multiple Reaction Monitoring (600 MRM, covering 14 classes of compounds in serum samples. The sample preparation followed the above procedure. An H-Class (Waters) UHPLC and utilized a Waters Atlantis Premier BEH Z-HILIC Column (1.7 μm, 2.1 mm^*^150 mm) for the chromatographic separation of target compounds. Chromatographic separation was achieved using mobile phase A (8:2 ultrapure water: acetonitrile with 10 mmol/l ammonium acetate) and mobile phase B (9:1 acetonitrile: ultrapure water with 10 mmol/l ammonium acetate). Both phases (A and B) were adjusted to a pH of 9 with ammonia. The sample tray temperature was set at 8°C, and a 1 μl injection volume was used. For mass spectrometric analysis in multiple reaction monitoring (MRM) mode, the project utilized a SCIEX 6500 QTRAP + triple quadrupole mass spectrometer equipped with an IonDrive Turbo V ESI ion source. The ion source parameters were as follows: Curtain Gas = 35 psi, IonSpray Voltage = +5000 V/−4500 V, Temperature = 400°C, Ion Source Gas 1 = 50 psi, Ion Source Gas 2 = 50 psi. The concentration of the target metabolite in the sample (C*_M_*, nmol/l) was calculated according to the equation presented above.

### Microbiome-wide associations and correlation analysis

Correlation analysis between differential rumen BAs and rumen microbial taxonomy and functions (KOs and CAZymes), as well as the internal correlation between rumen differential BAs, were conducted in the CB group and the BA-vivo group, respectively, as very rare BAs were found in the two control groups (Spearman’s rank correlation coefficient (|*r*|) = 1 being considered as significant, *n* = 5). The Spearman’s rank correlation with Benjamini–Hochberg correction was conducted between serum differential BAs and serum other different metabolites (adjusted *P* < .05 being considered as significant, *n* = 12). The Spearman’s rank correlation coefficient was calculated in the R project psych package. Network of these correlation coefficients was generated using the igraph package in the R project.

### Mediation analysis

This mediation analysis examined the mediating effect of the mediator on the association of the treatment with outcomes [44, 45]. The mediation analysis was performed using the R package “mediation” with consistent parameter settings (boot = “TRUE”, boot.ci.type = “perc”, conf.level = 0.95, sims = 1000). We conducted sensitivity analysis to assess the mediation effect’s robustness and examine the violation of the assumption (sequential ignorability) using the “medsens” R package with default parameters [46]. The presentation of mediation results adhered to the Guideline for Reporting Mediation Analyses (AGReMA) statement [47].

### Statistical analysis

The *in vitro* fermentation characteristics were evaluated using a one-way ANOVA in SPSS 26.0 (IBM, NY, USA). A Student’s *t*-test in SPSS 26.0 (IBM, NY, USA) was employed for analyzing all other fermentation parameters between the two groups. Statistical significance was declared at a *P* value <.05. Abundances of microbial metabolic pathways, KOs, and CAZymes, as well as rumen microbial domains, phyla, class, order, family, genera, and species, were compared using the Wilcoxon rank-sum test (*P* value <.05), indicating statistical significance. Differences in the Cytochrome P450 Engineering Database (CYPED) function were employed using MetagenomeSeq (*P* value <.05). The microbial taxonomy was also compared using linear discriminant analysis effect size (LEfSe, linear discriminant analysis (LDA) score > 2 and *P* value <.05) and using Analysis of Compositions of Microbiomes with Bias Correction (ANCOM-BC) [48] (false discovery rate < 0.05) to find the most crucial BA-associated rumen microbes. For metabolomics, normalized peak areas were input into the SIMCA16.0.2 software package (MKS Data Analytics Solutions, Umea, Sweden) for principal component analysis (PCA) and orthogonal projections to latent structures for discriminant analysis. The first principal component of variable importance in the projection (VIP) was used to refine the analysis results. Metabolites with VIP values exceeding 1.0, along with variables assessed by Student’s *t*-test with a *P* value <.05, were identified as differential metabolites.

## Results

### Integrating datasets of BA metabolism-associated microbiomes based on rumen MAGs analysis

**Figure 1:**
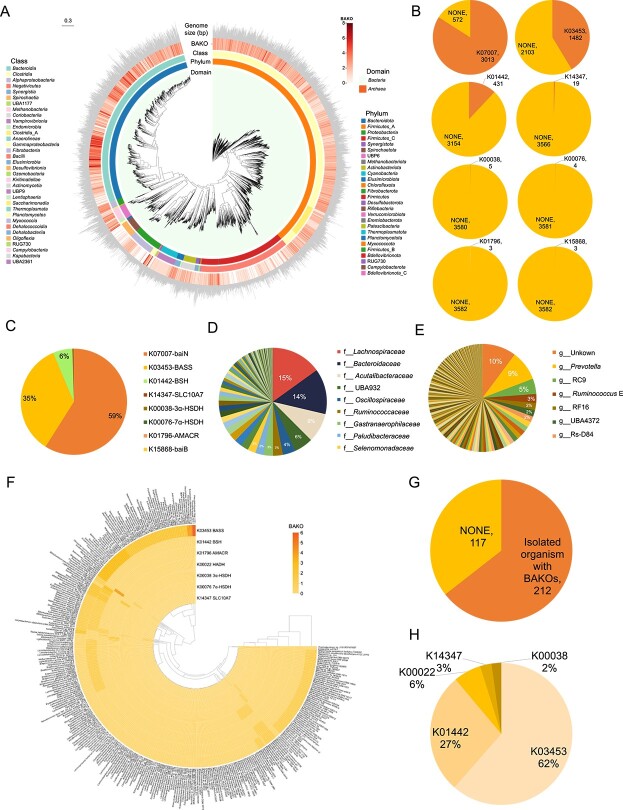
Phylogenetic tree of rumen metagenome-assembled genomes (MAGs) re-analysis. (**A**) The clades are colored according to domain. The charts represent the MAGs from different phylum and class-level affiliations of MAGs. The heat map indicate the number of bile acid metabolism associated KEGG ortholog (BAKO) in each MAG, including 3-dehydro-bile acid Delta4,6-reductase (*baiN*), bile acid:Na + symporter family (*BASS*), bile salt hydrolases (*BSH*), solute carrier family 10 (sodium/bile acid cotransporter), member 7 (*SLC10A7*), 3alpha(or 20beta)-hydroxysteroid dehydrogenase (*3α-HSDH*), *7α-HSDH*, bile acid-coenzyme a ligase (*baiB*, 0.06%), and alpha-methylacyl-CoA racemase (*AMACR*). The outer layer of the bar graph represents genome size. (**B**) The constructed database of the 8 BAKOs in the rumen MAGs. (**C**) The proportion of each BAKO in the BAKO gene database, showing that the primary BAKOs are K07007 (4623, 59%), K03453 (2716, 35%), and K01442 (466, 6%). (**D-E**) The main BAKOs carrying MAGs proportion at the taxonomy family level (**D**), genus level (**E**). (**F**) 7 BAKOs in 212 isolated rumen organisms: K00022 (3-hydroxyacyl-CoA dehydrogenase, HADH), K00038 (*3α-HSDH*), K00076 (*7α-HSDH*), K01442 (*BSH*), K01796 (*AMACR*), K03453 (*BASS*) and K14347 (*SLC10A7*). (**G**) The ratio of isolated organisms with BAKOs to isolated organisms without BAKOs (NONE). (**H**) Ratio among the 7 BAKOs annotated. The letters f and g appearing in front of taxa names denote the family and genus levels, respectively.

After filtering, a total of 8645 high-quality ruminal MAGs were obtained from 9371 rumen MAGs. Following the removal of redundancy, 3954 MAGs were selected for subsequent analysis. All 3954 MAGs were primarily represented by the “Bacteria” and “Archaea” (Fig. 1A). In this study, we defined 82 BAKOs as potential rumen BA metabolic enzymes (Supplementary Table S2), of which 15 had been previously identified as BAKOs in intestinal BA metabolism [49], including bile salt hydrolase (*BSH*), *7α-HSDH*, *7β-HSDH*, *12α-HSDH*, *12β-HSDH*, *3α-HSDH*, *3β-HSDH*, 3α-hydroxy BA-CoA-ester 3-dehydrogenase (*baiA*), BA-coenzyme A ligase (*baiB*), 3-oxocholoyl-CoA 4-desaturase (*baiCD*), BA 7alpha-dehydratase (*baiE*), BA CoA-transferase (*baiF*), *baiN*, 7β-hydroxy-3-oxochol-24-oyl-CoA 4-desaturase (*baiH*), and BA 7β-dehydratase (*baiI*). Out of 3954 MAGs, 3585 BAKO-carrying MAGs (BAMAGs) were identified, constituting 91% (Fig. 1A), and 923 of them have species names. We defined these 923 MAGs as BAMAGs-s, which means BAKO-carrying MAGs at the species level. However, only eight BAKOs were identified based on these ruminal MAGs (Fig. 1B). In addition, 3013 out of the 3585 BAMAGs, belong to K07007 (*baiN*, 84%), 1482 BAMAGs belong to K03453 (*BASS*, 41%), 431 BAMAGs belong to K01442 (*BSH*, 12%), 19 BAMAGs belong to K14347 (solute carrier family 10 (sodium/bile acid cotransporter), member 7, *SLC10A7*, 0.53%), 5 BAMAGs belong to K00038 (*3α-HSDH*, 0.14%), 4 BAMAGs belong to K00076 (*7α-HSDH*, 0.08%), 3 BAMAGs belong to K15868 (*baiB*, 0.06%), and 3 BAMAGs belong to K00038 (α-methylacyl-CoA racemase, *AMACR*, 0.08%) (Fig. 1B). For the 7840 BAKOs associated gene numbers, the top three BAKOs are K07007 (4623, 59%), K03453 (2716, 35%), and K01442 (466, 6%), consist of the core BA metabolic function (Fig. 1C). Among these BAMAGs, 3519 were “Bacterial,” and 66 were “Archaea” MAGs. *Lachnospiraceae* (15%), *Bacteroidaceae* (14%), and *Acutalibacteraceae* (9%) were the dominant families (Fig. 1D). At the genus level, *Prevotella* (9%) was prevalent (Fig. 1E). We initially identified enzymes in the rumen microbiome’s *bai* operon cluster, including *baiN*, *7α-HSDH*, *3α-HSDH, baiB*, and *BSH*, within rumen MAGs. This suggests potential bile acid biotransformation in the rumen, analogous to functions observed in humans [4].

### IMG/G rumen cultured organisms and functional gene annotation

We used the IMG system to screen 375 cultured microbial genomes from the rumen, 341 of which were downloadable. After removing duplicates, 329 isolated organisms were analyzed for BAKOs. A total of 212 organisms were annotated with 7 BAKOs: K00022 (3-hydroxyacyl-CoA dehydrogenase, *HADH*), K00038 (*3α-HSDH*), K00076 (*7α-HSDH*), K01442 (*BSH*), K01796 (*AMACR*), K03453 (*BASS*), and K14347 (*SLC10A7*). Specifically, annotations included 166 organisms for K03453, 74 for K01442, 17 for K00022, 7 for K14347, 6 each for K00038 and K01796, and 3 for K00076 (Fig. 1F–H). Only *HADH* differed from the previously analyzed rumen MAGs.

### Modification of rumen microbial anaerobic fermentation by exogenous BAs introduction *in vitro* and *in vivo*

**Figure 2:**
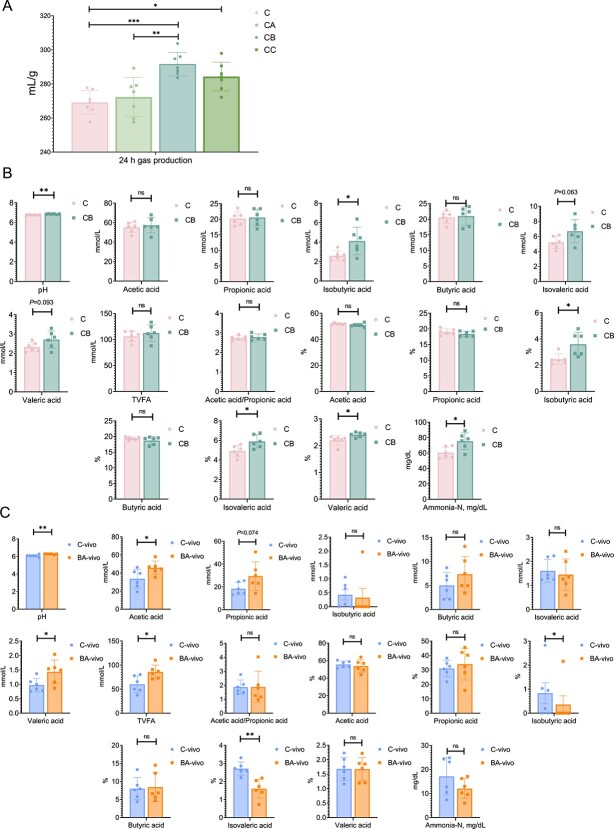
Rumen fermentation characteristic *in vitro* and *in vivo*. (**A**) The *in vitro* gas production under 0, 8, 16, 32 mg exogenous bile acids treatments (*n* = 7). (**B**) The 16 mg bile acids group exhibited significantly upregulated rumen pH and isobutyric acid concentration, and increased proportion of isobutyric acid, valeric acid, and isovaleric acid. (**C**) The significantly evaluated values of rumen pH, acetic acid, and total VFAs, and the proportions of acetic acid, valeric acid, and isovaleric acid by the BA-vivo group compared to the C-vivo group. Statistical differences were assessed by one-way ANOVA with Tukey’s test (A), and two-tailed unpaired Student’s *t*-test (B, C). Bars represent mean ± s.d. ^*^P < .05, ^*^^*^P < .01, ns, not significant.

In the preliminary *in vitro* anaerobic cultivation, compared to the C group, CB and CC groups exhibited a significantly increased gas production (Fig. 2A). An upregulated rumen pH, isobutyric acid concentration, and proportions of isobutyric acid, valeric acid, and isovaleric acid within the total VFA were found in the C group compared to the CB group (Fig. 2B). In the *in vivo* study, the concentrations of total VFA, acetate, and valerate were significantly elevated in the BA-vivo group compared to the C-vivo group (Fig. 2C). We found that both the *in vivo* and *in vitro* experiments significantly increased pH.

### Rumen BA profile *in vitro* and *in vivo*

**Figure 3:**
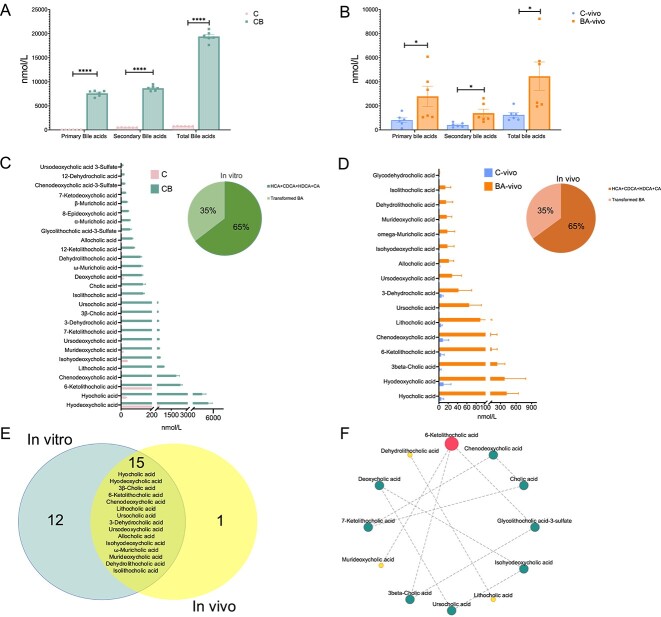
Rumen quantitative bile acid metabolomics analysis. (**A-B**) Exogenous BAs significantly alter ruminal primary, secondary, and total bile acid contents from *in vitro* (**A**) and *in vivo* studies (**B**). (**C-D**) The significantly changed individual bile acids from *in vitro* (**C**) and *in vivo* (**D**) studies. The concentration of new identified bile acids accounted for 35% for both the *in vitro* or *in vivo* study. (**E**) The 15 mutual differential bile acids present in both *in vitro* and *in vivo* experiments. (**F**) The internal spearman correlation analysis for these 15 different bile acids from the CB and BA-vivo groups individually (*n* = 6, spearman’s rank correlation coefficient (|*r*|) = 1). Statistical differences of were assessed by two-tailed unpaired Student’s *t*-test (*n* = 6). Bars represent mean ± s.d. ^*^P < .05, ^*^^*^P < .01, ^*^^*^^*^P < .001, ^*^^*^^*^^*^P < .0001.

PCA showed that the BAs were clearly separated from both *in vitro* and *in vivo* studies (Supplementary Fig. S2A and B). We observed a significant increase in primary BAs, secondary BAs, and total BAs in the CB group and BA-vivo group compared to the C group and the C-vivo group, respectively (Fig. 3A and B). In the *in vitro* experiment, we found 3 main primary BAs of HCA, CDCA, and CA; 4 new primary BAs like 3β-CA, α-MCA, β-MCA, and ω-MCA; and 20 secondary BAs, including HDCA, LCA, 6-ketoLDA, isoHDCA, MDCA, UDCA, 7-ketoLCA, DHCA, UCA, isolithocholic acid (isoLCA), DCA, DHLCA, 12-ketoLCA, alloCA, GlyLCA-3-sulfate, 3-epiDCA, 7-ketoDCA, CDCA-3-sulfate, 12-DHCA, and UDCA-3-sulfate were significantly different (Fig. 3C). In the *in vivo* study, the detected rumen BA pool was like the *in vitro* study with 16 significantly different compounds between the two groups (Fig. 3D). We found that the rumen-transformed BAs accounted for 35% of the *in vitro* CB group and the *in vivo* BA-vivo group (Fig. 2C and D). We identified 15 mutually increased BAs between the *in vitro* and *in vivo* trials, including UDCA, 6-ketoLCA, HDCA, HCA, 3-DHCA, 3β-CA, alloCA, LCA, UCA, MDCA, ω-MCA, isoHDCA, isoLCA, CDCA, and DHLCA (Fig. 3E). Subsequently, we conducted internal correlation analysis for these diverse BAs in the rumen from the CB and BA-vivo groups separately (*r* = |1|). 6-KetoLCA exhibited the highest number of correlations with other distinct BAs (Fig. 3F). These findings confirmed that the transformed compounds, including UDCA, 6-ketoLCA, 3-DHCA, 3β-CA, alloCA, LCA, UCA, MDCA, ω-MCA, isoHDCA, and DHLCA, represent new metabolic BAs distinct from the six originally added exogenous BAs.

### Rumen metagenomics analysis by exogenous BA introduction *in vitro* and *in vivo*

**Figure 4:**
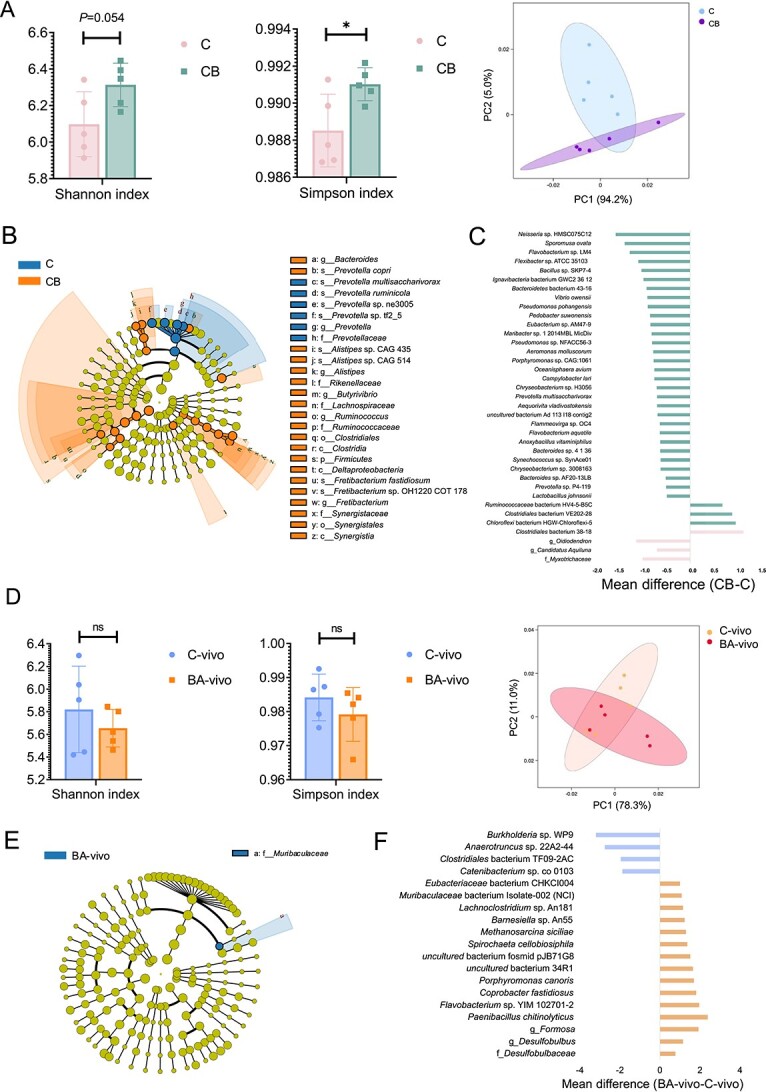
Metagenomics analysis of the rumen microbiome from *in vitro* study and *in vivo* study. (A) The administration of CB significantly affected the Simpson diversity index, with no significant impact on both the Shannon diversity index. Principal coordinates analysis (PCoA) reveals a distinct separation of rumen microorganisms between C and CB. (**B**) Linear discriminant analysis effect size (LEfSe) analysis between C and CB (linear discriminant analysis (LDA) score > 2 and *P* value < .05). (**C**) Analysis of compositions of microbiomes with bias correction (ANCOM-BC) between C and CB (false discovery rate < 0.05). (**D**) BA administration resulted in no significant impact on both Shannon diversity index and Simpson diversity index. PCoA reveals no apparent separation is observed between C-vivo and BA. (**E**) LEfSe analysis between C-vivo and BA (LDA score > 2 and *P* value < .05). (**F**) ANCOM-BC analysis between C-vivo and BA (false discovery rate < 0.05). The letters f, o, c, g, and s appearing in front of taxa names denote the family, order, class, genus, and species levels, respectively.

In the *in vitro* study, the administration of CB significantly impacted the Simpson diversity index (Fig. 4A). PCA further supported these findings by revealing an apparent separation of rumen microorganisms (Fig. 4A). The microbial domains indicated that “Bacteria,” “Fungi,” “Archaea,” “Viral,” and “Protozoa” species contribute the most to the assigned rumen microbiota, with unassigned microbiota also contributing nearly 20% (Fig. S3A and B). We found 8 different phyla, 5 class, 35 family, 162 genera, and 723 species in the *in vitro* study based on Wilcoxon rank-sum test (Table S3a). For differential abundance comparison analysis using LEfSe, the abundance of the *Firmicutes* was significantly higher in the CB group. At the family level, *Ruminococcaceae*, *Lachnospiraceae*, *Rikenellaceae*, and *Synergistaceae* were significantly higher in the CB group. At the genus level, *Bacteroides*, *Alistipes*, *Butyrivibrio*, *Ruminococcus*, and *Fretibacterium* were significantly higher in the CB group. At the species level, the abundance of *Prevotella copri*, *Alistipes* sp. CAG_435, *Alistipes* sp. CAG_514, and *Fretibacterium fastidiosum* were significantly higher in the CB group (Fig. 4B). For differential abundance comparison analysis using ANCOM-BC, the abundances of *Clostridiales* bacterium 38-18, *Chloroflexi* bacterium HGW-Chloroflexi-5, *Clostridiales* bacterium VE202-28, and *Ruminococcaceae* bacterium HV4-5-B5C were significantly higher in the CB group (Fig. 4C).

In the *in vivo* study, similar trends were observed in alpha-diversity and beta-diversity (Fig. 4D). Like the *in vitro* study, microbial domains, including “Bacteria,” “Fungi,” “Archaea,” “Viral,” and “Protozoa” species, showed no significant differences between BA-vivo and C-vivo (Supplementary Fig. S3C and D). We identified 4 phyla, 2 classes, 17 families, 92 genera, and 407 species that differed between BA and C-vivo (Supplementary Table S3b). For differential abundance comparison analysis using LEfSe, the abundance of the family *Muribaculaceae* was significantly higher in BA-vivo (Fig. 4E). For differential abundance comparison analysis using ANCOM-BC, the abundances of family *Desulfobulbaceae*, genera *Desulfobulbus* and *Formosa*, and species *Paenibacillus chitinolyticus*, *Flavobacterium* sp. YIM 102701-2, *Coprobacter fastidiosus*, *Porphyromonas canoris*, *uncultured* bacterium 34R1, *uncultured* bacterium *fosmid* pJB71G8, *Spirochaeta cellobiosiphila*, *Methanosarcina siciliae*, *Barnesiella* sp. An55, *Lachnoclostridium* sp. An181, *Muribaculaceae* bacterium Isolate-002, and *Eubacteriaceae* bacterium CHKCI004 were significantly higher in the CB group (Fig. 4F).

The three key BAKOs (*baiN*, *BASS*, and *BSH*) were consistently present in all samples and showed no significant differences between the C and CB groups or between the C-vivo and BA-vivo treatments (Supplementary Table S4a). In the *in vitro* study, we identified 169 altered KOs (Supplementary Table S4b), and in the *in vivo* study, 126 KOs exhibited changes (Supplementary Table S4c). In addition, an analysis utilizing the CYPED revealed an increase in the expression of *CYP107*, a cytochrome P450 monooxygenase, in the CB group compared to the C group (Supplementary Table S4d). Four mutual KOs (K19076: CRISPR-associated protein Cmr2; K13930: triphosphoribosyl-dephospho-CoA synthase; K03079: L-ribulose-5-phosphate 3-epimerase; K00853: L-ribulokinase), 1 mutual different carbohydrate-active enzymes (CAZymes, GH42: glycoside hydrolase family 42, β-galactosidases), 1 mutual different KEGG pathway (caprolactam degradation), 1 mutual family bacteria (*Desulfomicrobiaceae*), 5 mutual genus (*Falsibacillus*, *Longilinea*, *Fusibacter*, *Pyramidobacter*, and *Thermincola*), and 23 mutual species (such as *Lactobacillus paralimentarius*, *Pontibacter* sp. BAB1700, and *Massilimaliae massiliensis*) were found (Supplementary Fig. S3E and F).

By comparing the annotated family, genera, and species of the BAMAGs from both *in vitro* and *in vivo* studies, the families *Lactobacillaceae*, *Christensenellaceae*, *Atopobiaceae*, *Anaerolineaceae*, and *Muribaculaceaegenera*; the genera *Dorea*, *Olegusella*, *Corynebacterium*, *Mailhella*, *Olsenella*, *Parasporobacterium*, *Acetatifactor*, *Ruminococcus*, *Oribacterium*, *Pyramidobacter*, *Duodenibacillus*, and *Lactobacillus*; and the species *Bacillus licheniformis* and *Bifidobacterium merycicum* were selected as the ruminal BA metabolic bacteria (Supplementary Fig. S3G).

### Modification of rumen MAGs by exogenous BAs introduction

**Figure 5:**
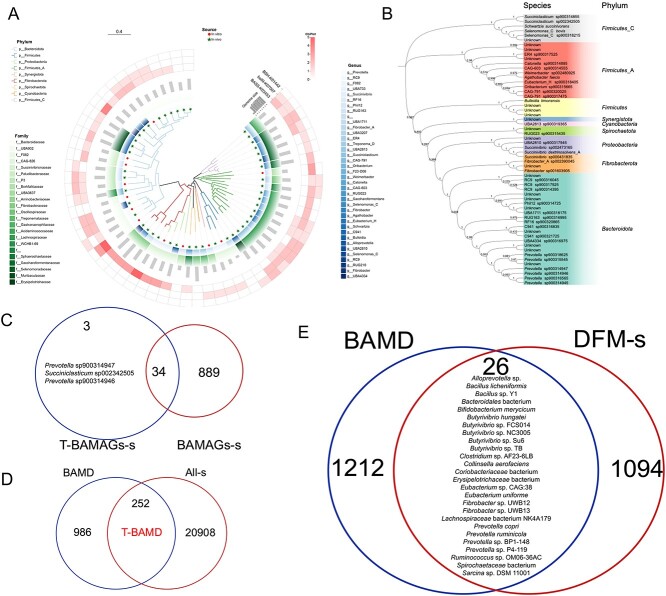
The modification of rumen metagenome-assembled genomes (MAGs) by exogenous bile acids introduction. (**A-B**) Phylogenetic tree of 58 MAGs were obtained from 20 rumen fluid samples from *in vitro* and *in vivo* studies. The MAGs annotated with phylum, family, genus (**A**), and species (**B**). The heat map indicate the number of three bile acid metabolism associated KEGG orthologs (BAKOs) in each MAG, including 3-dehydro-bile acid Delta4,6-reductase (*baiN*), bile acid:Na + symporter family (*BASS*), bile salt hydrolases (*BSH*). (**C**) Venn diagrams showing the 3 detected unclaimed rumen species after comparing rumen BAKO-carrying MAGs in species level (BAMAGs-s) with BAMAGs-s from this study (T-BAMAGs-s). (**D**) The constructed rumen BA metabolism microbiome database (BAMD) through rumen BAMAGs-s after remarking the GTDB species name with NCBI organism name as well as 212 isolated rumen organisms with BAKOs obtained from IMG/G, and the constructed BAMD from this study (T-BAMD) after comparing with all the detected microbes in species level (ALL-s). (**E**) Venn diagrams showing the 26 DFM-s (all different rumen microbes on species level by Wilcoxon rank-sum test (*P* value < .05), LEfSe (LDA score > 2 and *P* < .05), and ANCOM-BC (false discovery rate < 0.05)) with BAKO between BAMD and DFM-s from both *in vitro* and *in vivo* studies.

The metagenomic sequencing procedure generated 1 616 941 826 reads from 20 rumen fluid samples. After filtering out low-quality reads and those from host genes, the remaining data were assembled into 232 763 contigs, enabling the reconstruction of 104 MAGs with high quality. These MAGs, characterized by completeness over 80% and contamination below 10%, belonged to 9 bacterial phyla: *Bacteroidetes* (24 MAGs), *Firmicutes* (4 MAGs), *Firmicutes_A* (12 MAGs), *Firmicutes_C* (6 MAGs), *Fibrobacterota* (4 MAGs), *Proteobacteria* (4 MAGs), *Spirochaetota* (2 MAGs), *Synergistota* (1 MAG), and *Cyanobacteria* (1 MAG) (Fig. 5A). Of these, 39 BAMAGs were enriched with species names (Fig. 5B). Annotation analysis revealed the involvement of three key BAKOs (*BASS*, *baiN*, and *BSH*) in 52 MAGs consistent with the analysis of 9371 rumen MAGs. Among these, 10 MAGs (4 from C group, 6 from CB group) were from the *in vitro* study, and 42 MAGs (21 from C-vivo group, 21 from BA-vivo group) were from the *in vivo* study. Among these 52 MAGs, 37 MAGs were defined as BAMAGs-s from this study (T-BAMAGs-s). Comparing with the previously defined 923 rumen BAMAGs, three rumen BAMAGs were identified: *Prevotella* sp900314947, *Succiniclasticum* sp002342505 (NCBI organism name: *Acidaminococcaceae* bacterium), and *Prevotella* sp900314946 (Fig. 5C).

All BAMAGs with GTDB species names were matched with NCBI organism names through the constructed database (Supplementary Table S5a). All these BAMAGs, including the BAMAGs-s and T-BAMAGs-s, reconstructed with 1056 NCBI organism names, as well as 212 isolated rumen organisms with BAKOs obtained from IMG/G were treated as the constructed rumen BA metabolism microbiome database (BAMD). By comparing with the BAMD and all non-redundant genes set annotation species in this study, we detected 252 rumen species with BAKOs (Fig. 5D, Supplementary Table S5b), we defined them as T-BAMD. We defined DFM-s as all different rumen microbes on species level by Wilcoxon rank-sum test (*P* value < .05), LEfSe (LDA score > 2 and *P* value < .05), and ANCOM-BC (false discovery rate < 0.05). By comparing with the BAMD and DFM-s, we detected 26 different rumen species with BAKO function (Fig. 5E).

### Annotation of core BA metabolic-associated rumen microbiome

We found that *Bergeyella cardium*, *Butyrivibrio* sp. XPD2006, *Dermocarpellaceae*, *marine* bacterium AO1-C, *Oscillochloridaceae*, *Parabacteroides* sp. 203, *Sphingobacterium* sp. M46, *Stanieria*, K15855 (exo-1,4-beta-D-glucosaminidase), and K16203 (D-amino peptidase) were the core key microbiomes correlating with HDCA, isoHDCA, 6-ketoLCA, and MDCA (Supplementary Fig. S4A). *Fusibacter* sp. 3D3 emerged as the singular bacterium exhibiting mutual correlation between the *in vitro* and *in vivo* studies (Supplementary Fig. S4B). The *Bacteroides* sp. OF03-11BH*, Blautia* sp. AM47-4, *Eubacterium* sp. An11, *Eubacterium yurii*, *Faecalicatena orotica*, *Fusibacter* sp. 3D3, *Halobacteriovorax* sp. DA5, *Massilimaliae massiliensis*, and *Tissierellia* bacterium KA00581 were the core microbes based on their mutual set between correlation analysis and mutual different species from *in vitro* and *in vivo* studies (Supplementary Fig. S4C). The core microbes, namely *B. licheniformis*, *B. merycicum*, *Butyrivibrio hungatei*, and *Butyrivibrio* sp. NC3005. *Eubacterium uniforme* and *Sarcina* sp. DSM 11001 were identified based on their shared presence in both the correlation analysis and the differential species carrying BAKO (Supplementary Fig. S4C).

### Rumen BA biotransformation

**Figure 6:**
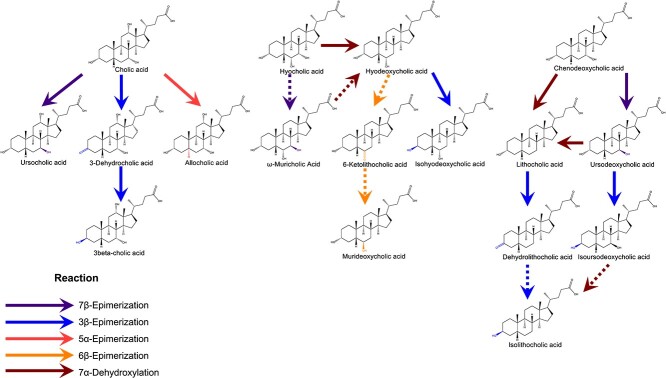
The proposed biotransformation pathway outlines the interactions between the 15 distinct bile acids and the modulated rumen microbes and enzymes. Direct simplified proposed pathway among the 15 distinct bile acids via five reactions including 7β-epimerization, 3β-epimerization, 5α-epimerization, 6β-epimerization, and 7α-dehydroxylation.

Based on previous studies and BA structures identified in the rumen, we determined the transformations between the following BAs: UCA is a metabolite of CA via 7β-epimerization [50]. 3-DHCA is a metabolite of CA via 3β-epimerization [51]. AlloCA is an isomer of CA via 5α-epimerization [52]. HDCA is produced from HCA via 7α-dehydroxylation [53]. IsoHDCA is a 3β epimer of HDCA via 3β-epimerization [54]. For CDCA, it has been demonstrated along the following pathways: CDCA→LCA [55], CDCA→UDCA [56], UDCA→LCA [57], LCA→DHLCA [58], and UDCA→isoUDCA [59]. In addition, based on the structural characteristics of BAs, we speculate that the BA metabolism pathways that occur in the rumen may be as follows. Because ω-MCA has been proven to produce HCA and further produce HDCA through isomerization of the C7 position [60], we speculate that HCA can produce ω-MCA through 7β-epimerization, whereas ω-MCA can produce HDCA through 7α-dehydroxylation. At the same time, we speculate that DHLCA generates isoLCA through 3β-epimerization, and IsoUDCA generates isoLCA via 7α-dehydroxylation (Fig. 6). After the exogenous BAs introduction, we found 24 and 7 different *Clostridium* spp. from *in vitro* and *in vivo* studies, respectively (Supplementary Table S6a and b).

### Reshaped circulating serum BA pool and host metabolism

**Figure 7:**
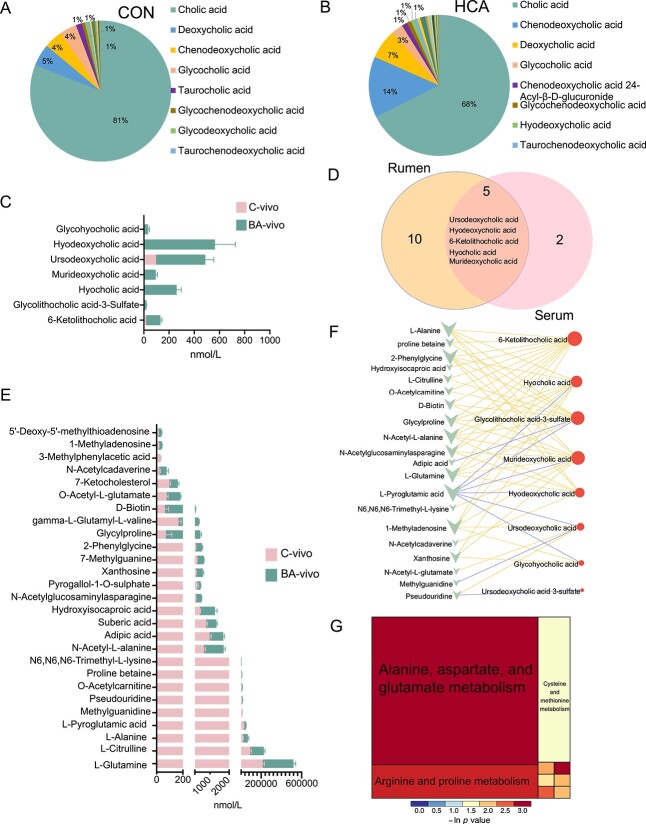
Serum bile acid pool and association with host metabolism. (**A-B**) The blood circulating bile acid pool in sheep is predominantly composed of cholic acid (CA), chenodeoxycholic acid (CDCA), and deoxycholic acid (DCA) from C-vivo (**A**) and BA (**B**). (**C**) The significantly upregulated bile acids in the serum by the BA-vivo group by targeted bile acid metabolomics. (**D**) The mutual differential bile acids between rumen and serum. (**E**) The targeted serum 600 compounds metabolomics (600C) analysis identified various substances, including amino acids, nucleic acids, organic acids, steroids, and others. (**F**) Association of the serum differential bile acids with differential 600C compounds (adjusted *P* < .05, *n* = 12). (**G**) The enriched metabolic pathways based on the detected differential serum compounds. Data are mean ± s.d.; statistical differences were assessed by variable importance in the projection (VIP) > 1.0 and Student’s *t*-test with a *P* value < .05 (*n* = 6).

The exogenous BAs could not change the serum top 4 dominant BA pool (Fig. 7A and B), but significantly upregulated serum values of HDCA, UDCA, HCA, 6-ketoLCA, MDCA, GlyHCA, and GlyLCA-3-sulfate by the BA-vivo group (Fig. 7C). We found that HDCA, UDCA, HCA, 6-ketoLCA, and MDCA were the mutually differential BAs between rumen and serum (Fig. 7D). In our analysis of the 600C metabolomics database, we detected 262 valid compounds. Twenty compounds exhibited higher abundance, whereas seven compounds displayed lower abundance in the BA-vivo group (Fig. 7E). The BA-vivo group demonstrated higher levels of L-glutamine, L-citrulline, and L-alanine, ranking as the top three compounds with the highest concentrations. Conversely, L-pyroglutamic acid and methylguanidine, the top two highest concentrations, were lower in the HCA group. Correlations were identified between changes in these differential compounds and variations in serum BAs, such as L-pyroglutamic acid and 6-ketoLCA (Fig. 7F). Differential metabolites in serum exhibited significant enrichment in pathways related to alanine, aspartate, and glutamate metabolism, D-glutamine and D-glutamate metabolism, and arginine and proline metabolism, indicating that BA metabolism in the rumen can affect the amino acid metabolism of the host (Fig. 7G).

### Mediation analysis among the rumen microbiome and host metabolism

**Figure 8:**
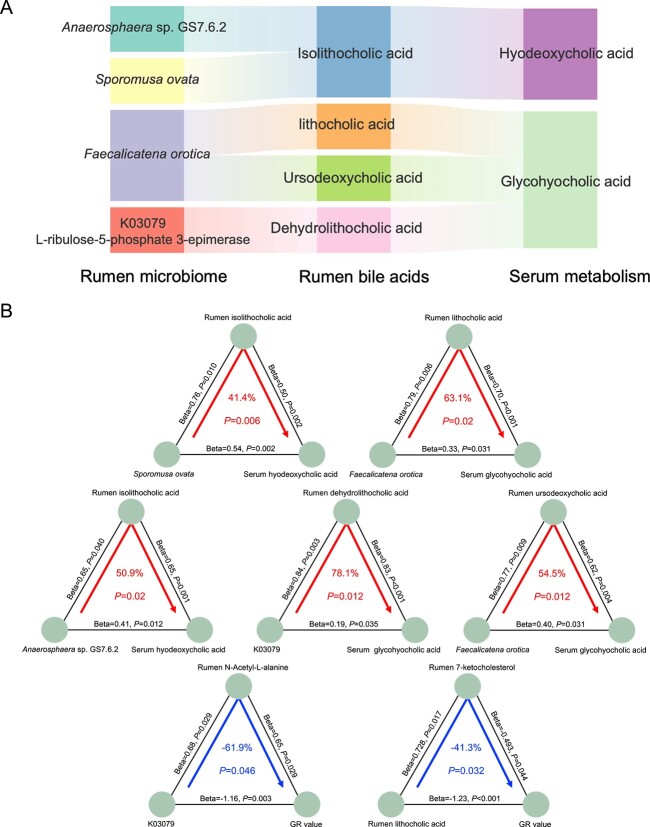
Rumen microbiome-dependent bile acids contributions to host metabolism via mediation analysis. (**A**) Parallel coordinates chart showing the mediation effects among rumen microbiome (left), rumen bile acids (middle) and serum metabolism (right). (**B**) The positive-related microbial biomarker *Sporomusa ovata*, *Faecalicatena orotica*, *Anaerosphaera* sp. GS7.6.2, *Faecalicatena orotica*, and K03079 (L-ribulose-5-phosphate 3-epimerase) affect the serum BA profiles through specific rumen BA biomarkers (such as isolithocholic acid and lithocholic acid). The inverse-related microbial biomarker of K03079 and lithocholic acid affect the host GR value (subcutaneous fat deposition) through specific serum metabolites biomarkers of N-acetyl-L-alanine and 7-ketocholesterol, respectively. The lines connect each two items indicate the associations with corresponding normalized beta values and *P* values. The arrowed lines connect three items indicate the microbial effects on host metabolism mediated by specific biomarkers, with the corresponding mediation *P* value. A *P* value <.05 is considered significantly different.

We conducted mediation analysis to identify mediators in the relationship between the rumen microbiome and the host (Fig. 8A and B). Specifically, rumen isoLCA emerged as a mediator in the positive association between *Anaerosphaera* sp. GS7.6.2 and serum HDCA, contributing to 50.9% of the mediation effect (*P*_mediation_ < 0.01). Similarly, rumen isoLCA mediated the positive relationship between *Sporomusa ovata* and serum HDCA, with a mediation effect of 41.4% (*P*_mediation_ < 0.05). Additionally, we observed that rumen LCA mediated the positive association between *Faecalicatena orotica* and serum GlyHCA, accounting for 63.1% of the mediation effect (*P*_mediation_ < 0.05). Furthermore, rumen UDCA acted as a mediator in the positive relationship between *Faecalicatena orotica* and serum GlyHCA, contributing to 54.5% of the mediation effect (*P*_mediation_ < 0.05). Rumen DHLCA was identified as a mediator in the positive association between K03079 and GlyHCA, accounting for 78.1% of the mediation effect (*P*_mediation_ < 0.05). These findings underscore the crucial role of ruminal microbial BAs in the interplay between the ruminal microbiome and host blood metabolome. Furthermore, we observed that serum N-acetyl-L-alanine mediated the inverse association of K03079 with the GR value (−61.9%, *P*_mediation_ < 0.05), and serum 7-ketocholesterol mediated the inverse association of rumen LCA with the GR value (−41.3%, *P*_mediation_ < 0.05).

## Discussion

Elucidating the biological characteristics of gut bacteria with the capacity to convert primary host BAs into secondary BAs holds particular importance, given the substantial impact of the microbial BA metabolites on the modulation of microbiome-host interactions [2]. The impact of the rumen microbiome on BA metabolism is largely unknown. We investigated the role of unreported BA metabolic enzymes and microbes in the rumen, shedding light on potential BA metabolic activity and characterizing their association with BA biotransformation. Excluding the externally introduced BAs, it was observed that primarily HDCA and 6-ketoLCA were newly synthesized by microbial processes in the rumen. This finding demonstrates that the rumen microbiome might contribute to the production of functional secondary BAs.

In the context of BA metabolism in the rumen, our study illuminates the dynamic nature of microbial capabilities to synthesize these compounds. These transformations occurred through five distinct reactions, including 7β-epimerization, 3β-epimerization, 5α-epimerization, 6β-epimerization, and 7α-dehydroxylation, as documented in prior studies [4, 61, 62]. 5α and 6β-epimerization, found in the prokaryotic and eukaryotic cells [61, 62], were found in the rumen. It is commonly accepted that the *bai* operon, which contains eight gene clusters (*baiBCDEAFGHI*) contributes to the 7α-dehydroxylation pathway, such as the transformation of CA to DCA [2]. The microbial enzymes outside the *bai* gene cluster also play a role in the 7α-dehydroxylation reaction. Moreover, it is particularly notable that HDCA emerges as a metabolite from the metabolic pathways of multiple primary BAs [63]. Thus, we estimated that HDCA was derived from BA via the 7α-dehydroxylation pathway in the rumen, but with a different mechanism. Additionally, LCA can be formed from CDCA by the enzymatic processes of intestinal bacteria in the human microbiome [4, 5]. UDCA can also be formed from CDCA by enzymatic processes [4]. Currently, over 20 types of carbohydrate epimerases have been documented. These enzymes exhibit distinct specificities, recognizing carbohydrate substrates at positions C1, C2, C3, C4, C5, or C6, such as D-ribulose-5-phosphate 3-epimerase, L-ribulose-5-phosphate 4-epimerase, UDP-galactose 4-epimerase, dTDP-6-deoxy-D-xylo-4-hexulose 3,5-epimerase, GDP-mannose 3,5-epimerase, and ADP-L-glycero-D-mannoheptose 6-epimerase [64]. We identified K03079 (L-ribulose-5-phosphate 3-epimerase) as a mutually different microbiome function in both *in vitro* and *in vivo* studies. Consequently, we propose that L-ribulose-5-phosphate 3-epimerase may represent an enzyme contributing to the process of 3β-epimerization in BA biotransformation within the rumen. One isoform of *CYP107*, specifically *CYP107D1* (*OleP*), has been documented to hydroxylate LCA, yielding MDCA as the sole product [65]. Concurrently, we observed elevated concentrations of LCA and MDCA in the CB group relative to the C group. This finding suggests the presence and metabolic involvement of *CYP107* in the rumen, particularly in the conversion of LCA to MDCA. The incidence of these BA biotransformations was facilitated by the rumen microbiome, emphasizing the unique characteristics inherent to the rumen microbial community.

The metabolism of secondary BAs is a dynamic process marked by variations in the microbial capacity to synthesize these secondary BAs. We found many ruminal microbial species were enriched after exogenous BAs introduction. *Clostridium* can perform oxidation and epimerization of hydroxy groups at the positions C3, C7, and C12 of bile salts, generating isobile (β-hydroxy) salts [66]. It was found that gram-positive bacteria from the *Clostridiales* order, such as *Ruminococcaceae*, are capable of performing 7α-dehydroxylation to transform primary BAs into secondary BAs [66]. *Fusibacter* sp. 3D3 was included in the arsenic metabolism, and it harbors ferredoxin-NAD+ oxidoreductase and electron transfer flavoprotein-coding genes [67]. It was also characterized that the flavoprotein was involved in the “reductive arm” of the microbial BA 7-dehydroxylation pathway [68]. Thus, *Clostridiales* and *Fusibacter* sp. 3D3 might have the 7-dehydroxylation capacity that contributes to the BA transformation in rumen.

The inherent BA tolerance and secondary BA metabolic capabilities of rumen microbes may have existed but were previously overlooked. The capacity for bile salt tolerance is a critical parameter in evaluating the potential probiotic functionality of microbes [69]. The metabolic processing of particular BAs may play a role in diminishing the susceptibility to pathobiont infections, which in turn could be pivotal in upholding gut homeostasis and host health [70]. *Butyricicoccus pullicaecorum*, a butyrate producer with probiotic potential, showed a correlated positive with *baiN* and had bile tolerance in terms of viability and metabolic activity [71]. *B. licheniformis* has been documented to serve as a probiotic in therapeutic interventions for both human and animal diseases, exerting anti-inflammatory and immunostimulatory effects, contributing to the regulation of lipid profiles [72]. Additionally, high serum 7-ketocholesterol levels led to acute myocardial infarction, an increase in the number of affected vessels, and high sensitive C-reactive protein concentrations in the subjects with coronary artery disease, indicating the association of circulating 7-ketocholesterol with cardiovascular outcomes [73]. Serum glycylproline is positively correlated with liver events in the late stages of non-alcoholic fatty liver disease [74]. Methylguanidine is a uremic toxin and marker of renal failure [75]. It is still unclear whether BA metabolism in the rumen has an impact on host health based on the changed serum metabolome in the current study, arguably through this BA-mediated rumen-host bidirectional biological process. Even though the exogenous BAs did not alter the detected blood immune function-related parameters, the changes in serum 7-ketocholesterol, glycylproline, and methylguanidine observed in this study confirm the BAs’ potential roles in enhancing health and using HCA to reduce inflammatory responses [16]. However, the changed serum levels of 7-ketocholesterol, glycylproline, and methylguanidine from this study confirmed the BAs’ potential health-enhancing roles and the use of HCA to reduce inflammatory responses.

Citrulline, primarily derived from the conversion of glutamine in the enterocyte, serves as an indicator of the functional enterocyte metabolic mass, including the small bowel, which is excluded from the digestive circuit [76]. The elevated serum L-citrulline levels in the BA-vivo group may be associated with the impact of the BA-vivo group on amino acid catabolism. These findings suggest that the BA-vivo group has discernible effects on amino acid metabolism. Additionally, we observed changes in the content and proportion of isobutyric acid and isovaleric acid in the rumen from both *in vitro* and *in vivo* studies. It has been reported that branched-chain fatty acids can be utilized by rumen bacteria to synthesize branched-chain amino acids [77]. Therefore, beyond the well-documented conjugation and deconjugation interactions between amino acids and BAs mediated by gastrointestinal microorganisms [4], the potential impact of BAs on amino acid metabolism in the rumen also merits further exploration.

HDCA could alleviate non-alcoholic fatty liver disease by enhancing lipid catabolism [15]. The UDCA had efficient roles in the treatment of obesity and alleviating metabolic dysfunction [78]. It has been postulated that the consumption of dietary L-pyroglutamic acid may elicit favorable alterations in glucose and lipid metabolism in diabetic rats and mice, thus potentially contributing to the amelioration of type 2 diabetes mellitus [79]. In addition, alanine is recognized as the principal amino acid released from muscle, which regulates inter-organ glucose homeostasis via the glucose-alanine cycle [80]. Therefore, the changes in serum alanine and pyroglutamic acid in the BA-vivo group may also be related to glucose metabolism. Biotin acts as a crucial cofactor for carboxylases, playing essential roles in fatty acid synthesis and mitochondrial oxidation, particularly within human adipose tissue [81]. Previous studies have associated suberic acid with a reduction in obesity, the regulation of blood lipid levels, a decrease in fat accumulation in liver cells, and the mitigation of lesions in rat cardiac arteries [82]. Similar to suberic acid, the concentration of adipic acid also appears to influence the lipid phenotype in the BA-vivo group, possibly involving suberic acid-related metabolic pathways [83]. Therefore, we posit that BA metabolism in the rumen may impact the levels of host serum biotin, suberic acid, and adipic acid, thereby influencing or reflecting the changed lipid metabolism. Collectively, our findings provide evidence that altered BA metabolism has repercussions on glucose, amino acid, and lipid metabolism, as elucidated by the serum metabolome.

It was reported that the composition of the *in vitro* headspace gas influences rumen fermentation outcomes, as evidenced by variations in total gas production and methane concentrations between N_2_ and CO_2_ headspaces; however, these conditions did not affect *in vitro* digestibility or the VFA profile [84]. Our experiment, meanwhile, is designed to assess the effects of BAs on rumen fermentation under the same conditions, thereby affirming the reliability and quality of our findings. Caution is advised when comparing absolute values across different studies. Additionally, the rumen fluid sampling in our *in vivo* studies used the liquid phase portion of the rumen contents, which may result in a higher proportion of microbiome and BAs in the liquid phase of the rumen fluid. However, exogenous BAs, as a small-molecule substance, mostly exist in the rumen liquid phase rather than adhering to feed particles and interacting with microorganisms in the liquid phase. The phase section might be a better choice. In future studies, BA metabolism in the solid fraction of the rumen can be studied in greater depth, such as how BAs interact with the rumen microbiome to influence the digestion of roughage in the rumen.

## Conclusions

In this study, our findings strongly imply that the rumen possesses the capacity to convert primary BAs into secondary BAs. We identified newly synthesized BAs and associated metabolic microbiomes within the rumen, suggesting that the rumen microbiome plays an integral role in the metabolism of BAs. Specifically, the microbiome appears to convert primary BAs, such as HCA and CDCA, into functional secondary or derived BAs, including HDCA, UDCA, LCA, and 6-ketoLCA. These transformed secondary BAs may interact with the rumen microbiome or enter the peripheral blood circulation directly, influencing host metabolism in glucose, amino acids, and lipids and facilitating a complex crosstalk between the host and the rumen microbiome. This highlights the pivotal role of the rumen microbiome in BA biotransformation, underscoring its significant impact on the host. The identification and characterization of BA-associated microbial sequences in the rumen provide a foundation for the targeted isolation of specific rumen microbes or functional microbial genes. Such isolation enables subsequent experiments to validate their roles in BA metabolism. Our findings are pivotal in developing therapies targeting the gut microbiome using rumen-derived BAs and microorganisms, offering promising future opportunities for treating metabolic disorders.

## Supplementary Material

Rumen_microbiome-driven_supplementary_materials-Rev03_wrae098

Supplementary_Tables-Final_wrae098

## Data Availability

The publicly available data that we re-analyzed here were generated by Xue *et al.* [18] (NCBI, PRJNA730102), Xie *et al.* [17] (European Nucleotide Archive, PRJNA657473), Glendinning *et al.* [19] (European Nucleotide Archive, PRJEB34458), Wilkinson *et al.* [20] (European Nucleotide Archive, PRJEB39057), Anderson *et al.* [21] (https://doi.org/10.6084/m9.figshare.12164250), and Stewart *et al.* [22] (European Nucleotide Archive, PRJEB31266). A summary of the dataset’s basic information and the corresponding accession numbers is available in Supplementary Table S7. The rumen metagenomic data from this study were uploaded to the NCBI Sequence Read Archive (SRA) under the accession number PRJNA1029673. The fasta files of all 104 rumen MAGs were deposited into the NCBI BioProject under the accession number PRJNA1045281.
