# Supplementary material for: Rumen microbiome-driven insight into bile acid metabolism and host metabolic regulation
Source: ISME J. 2024 Jun 5;18(1):wrae098. doi: 10.1093/ismejo/wrae098 (PMC11193847; doi:10.1093/ismejo/wrae098)
Supplement: Rumen_microbiome-driven_supplementary_materials-Rev03_wrae098 [file rumen_microbiome-driven_supplementary_materials-rev03_wrae098.docx]

Supplementary Materials for

**Rumen microbiome-driven insight into bile acid metabolism and host metabolic regulation**

Boyan Zhang *et al.*

*Corresponding author. Bing Wang, Email: [wangb@cau.edu.cn](mailto:wangb@cau.edu.cn)

**This PDF file includes:**

Fig. S1 to S4

Tables S1 to S7

|  |
| --- |
| Fig. S1.  The schematic overview of the workflow in this study. |

| 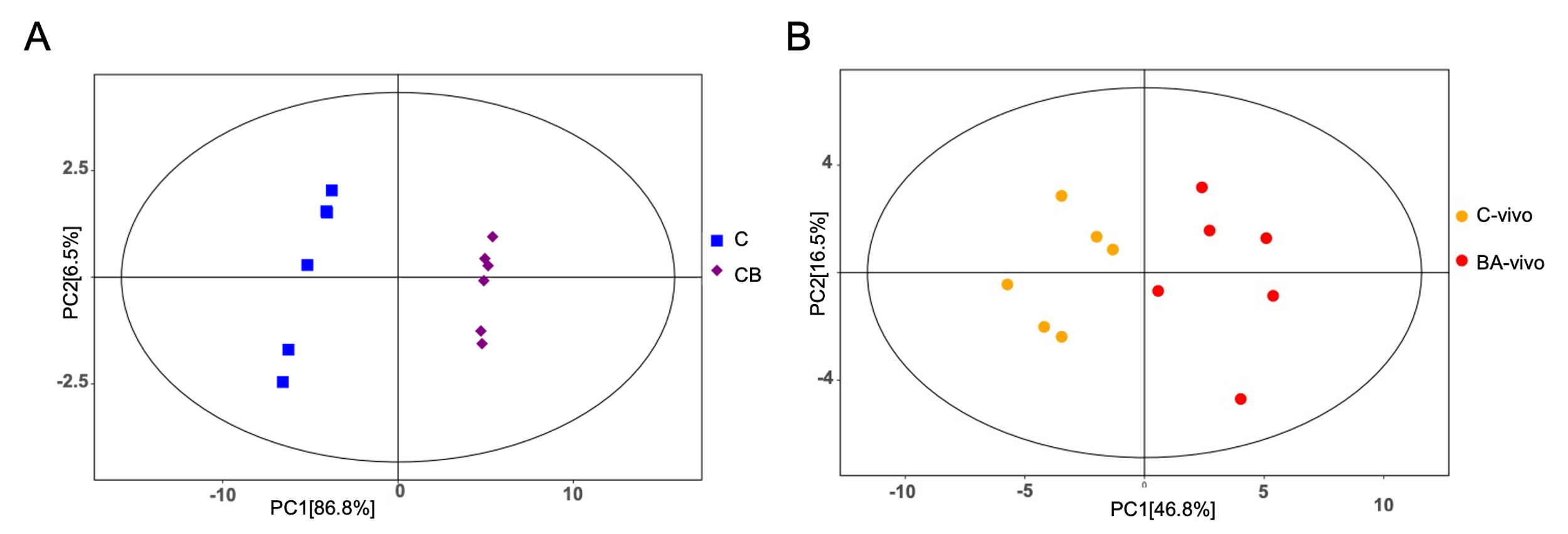 |
| --- |
| Fig. S2.  The metabolomics statistical analysis. (**A-B**) Principal component analysis (PCA) reveals a distinct separation of rumen bile acids in both the *in vitro* (**A**) and *in vivo* (**B**) studies. |

| 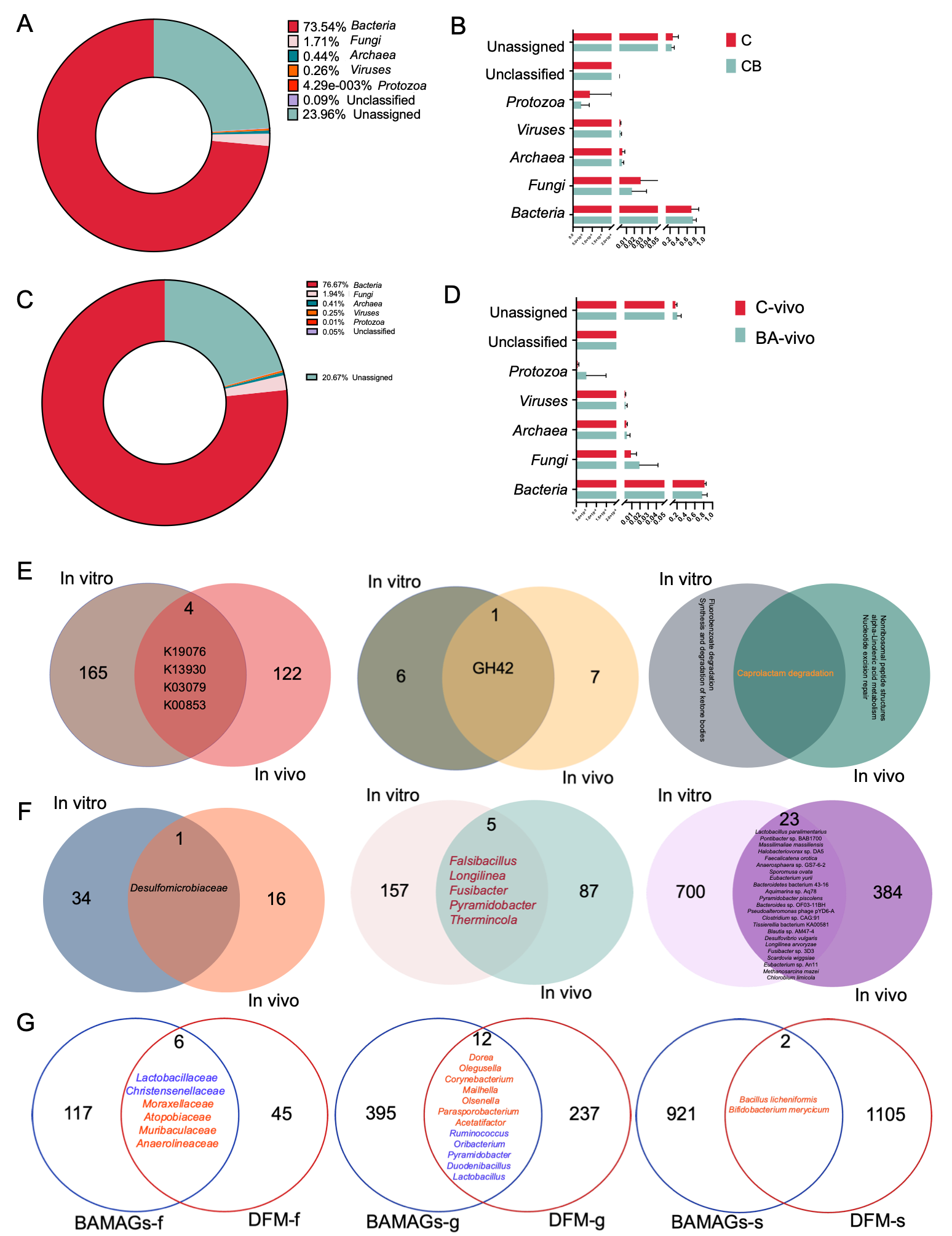 |
| --- |
| Fig. S3.  The microbial domains found in bacteria, fungi, archaea, viral, eukaryotic species, and unassigned microbiota from in vitro (**A**) and in vivo (**B**) studies, but not significantly differences were found between the C and CB groups (**C**), as well as C-vivo and BA-vivo groups (**D**). **(E)** Venn diagrams showing the 4 mutual KOs (K19076: CRISPR-associated protein Cmr2; K13930: triphosphoribosyl-dephospho-CoA synthase; K03079: L-ribulose-5-phosphate 3-epimerase; K00853: L-ribulokinase), 1 mutual different carbohydrate-active enzymes (GH42: glycoside hydrolase family 42, β-galactosidases), 1 mutual different KEGG pathway (caprolactam degradation) between the in vitro and in vivo studies. **(F)** Venn diagrams showing the 1 mutual family level microbes, 5 mutual genus level microbes, and 23 mutual species between the *in vitro* and in vivo studies. **(G)** Venn diagrams showing the potential BA metabolic microbes by the comparison between the bile acid metabolism KEGG ortholog carrying metagenome-assembled genomes (BAMAGs) and different microbes (DFM) at the family, genus, and species level. |

|   Fig. S4.  **Association of rumen microbiome structure with bile acid profile to identify the core rumen microbiome involved in bile acid metabolism. (A)** Spearman correlation analysis revealed a significant correlation between the core rumen bile acids (15 individual BAs, primary BAs, secondary BAs) and the distinct rumen microbiome, encompassing microbial taxonomy at the family, genus, and species levels, as well as functions (KEGG ortholog and carbohydrate-active enzymes). The analysis was conducted using samples from the CB (n = 5) and HCA (n = 5) groups (spearman’s rank correlation coefficient (\|r\|) = 1). **(B)** Venn diagrams illustrate the shared and correlated bacteria, specifically *Fusibacter* sp. 3D3, between the *in vitro* and *in vivo* correlation studies. (**C)** Venn diagrams showing the core microbes based on the mutual set among the correlated different species (Cor-DFM-s), and mutual different species from *in vitro* and *in vivo* studies (Mutual DFM-s), and BAKO carrying differential species (BA-DFM-s). |
| --- |

**All supplementary tables were integrated into a separate Excel file.**

Supplementary Tables.

**Table S1a**. Ingredients and nutrient composition of the basal diet fed for the animals from the *in vitro* study. **Table S1b**. Ingredients and nutrient composition of the basal diet fed for the animals from the *in vivo* study. **Table S2**. The defined potential 82 bile acid metabolism KEGG orthologs in this study. **Table S3a**. Different rumen microbial taxonomy from *in vitro* study. **Table S3b**. Different rumen microbial taxonomy from the *in vivo* study. **Table S4a**. The three-bile acid metabolism associated KEGG orthologs from the *in vitro* and *in vivo* studies. **Table S4b**. Different KEGG ortholog from the *in vitro* study. **Table S4c**. Different KEGG ortholog from the *in vivo* study. **Table S4d.** Differential expression of cytochrome P450 monooxidase as identified by MetagenomeSeq in an *in vitro* study. **Table S5a**. Species database construction for matching GTDB taxonomy with NCBI organism names. **Table S5b**. The detected different rumen microbes in species level with potential bile acid metabolism function. **Table S6a**. Different *Clostridium* spp. from the *in vitro* study. **Table S6b**. Different *Clostridium* spp. from the *in vivo* study. **Table S7**. A summary of the dataset’s basic information and the corresponding accession numbers.
